# Virus Movements on the Plasma Membrane Support Infection and Transmission between Cells

**DOI:** 10.1371/journal.ppat.1000621

**Published:** 2009-11-26

**Authors:** Christoph J. Burckhardt, Urs F. Greber

**Affiliations:** Institute of Zoology, University of Zürich, Zürich, Switzerland; The Scripps Research Institute, United States of America

## Abstract

How viruses are transmitted across the mucosal epithelia of the respiratory, digestive, or excretory tracts, and how they spread from cell to cell and cause systemic infections, is incompletely understood. Recent advances from single virus tracking experiments have revealed conserved patterns of virus movements on the plasma membrane, including diffusive motions, drifting motions depending on retrograde flow of actin filaments or actin tail formation by polymerization, and confinement to submicrometer areas. Here, we discuss how viruses take advantage of cellular mechanisms that normally drive the movements of proteins and lipids on the cell surface. A concept emerges where short periods of fast diffusive motions allow viruses to rapidly move over several micrometers. Coupling to actin flow supports directional transport of virus particles during entry and cell-cell transmission, and local confinement coincides with either nonproductive stalling or infectious endocytic uptake. These conserved features of virus–host interactions upstream of infectious entry offer new perspectives for anti-viral interference.

## Introduction

The plasma membrane is a highly dynamic organelle and fences off pathogens with considerable efficiency. Besides segregation, it coordinates cell migration, information processing, and endo- and exocytosis during signalling and homeostasis. It also transmits information between neighboring cells or cells at a distance. Viruses take advantage of the plasma membrane in various ways. They bind to attachment factors, move laterally, and interact with secondary signalling receptors, or engage into endocytosis or fusion with the plasma membrane. All of these events determine if a particular cell gets infected or resists against the pathogen. For many viruses, the interactions with attachment factors and receptors are well characterized, and endocytic pathways have been mapped and in part integrated with cell signalling (for a review, see [1]). Only recently, however, attention has been focussed on lateral motions of viruses at the plasma membrane prior to uptake [2],[3].

## Three Conserved Virus Motions Revealed by Single Virus Tracking and Trajectory Segmentation

Motions of single fluorescently labelled viruses on the plasma membrane are typically recorded with total internal reflection or confocal microscopy at high temporal resolution [4],[5]. Virus trajectories can be determined by powerful single particle tracking algorithms at subpixel resolution. The considerable heterogeneity of motions on the surface together with high temporal acquisition frequency require accurate and reliable processing of large datasets [6],[7],[8]. This allows the determination of overall properties of the trajectories, such as diffusion coefficients, mean square displacements, or moment scaling spectrum [9].

**Figure 1:**
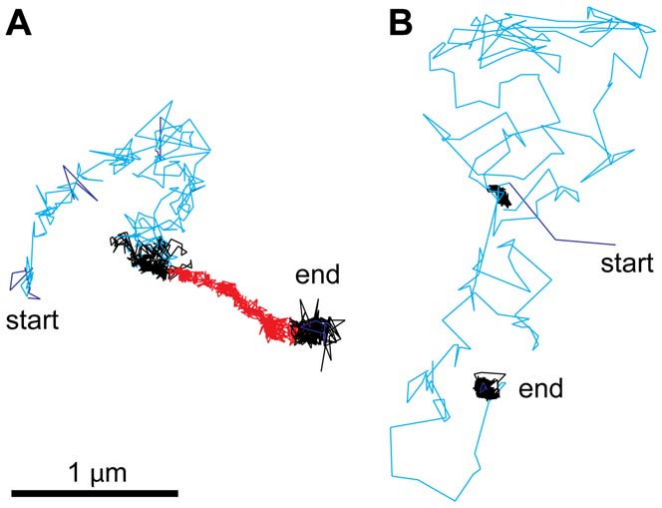
Diffusional motions cover larger surface areas than directed drifts and confined motions. Viruses have been observed to undergo three types of motion, random diffusion (cyan), retrograde drifts (also called retrograde flow, red), and confined motions (black) (see Table 1 and main text). (A and B) show the heterogeneity of two typical trajectories of adenovirus serotype 2 particles on human embryonic retinoblasts. The motion patterns were recorded by confocal microscopy at 25 Hz acquisition frequency and automatically classified by a machine-based learning algorithm [11]. Nonclassified motions are depicted in dark blue.

There is, however, more information in the movement patterns of virus particles at the plasma membrane, as indicated by the heterogeneity of individual trajectories [10]. The development of support vector machines for trajectory segmentation has recently allowed researchers to automatically identify trajectory fingerprints, including diffusive motions, drifting motions, and confinement [11] (see Figure 1A and 1B). These three motion types can be found with most of the viruses analyzed (Table 1). This suggests that diffusion, drifts, and confinements are general features of virus–host interactions that are driven by inherent properties of the plasma membrane rather than specific features of particular viruses.

**Table 1:** Viruses, Receptors, and Cell Surface Movements.

Virus	Family	1° Receptor	2° Receptor	Surface Motion	References
Avian leukosis virus (ALV)	Retroviruses	Low density lipoprotein receptor family members TVA (ALV-A subgroup), TVB (ALV-B), TVC (ALV-C)	?	Virus entry: Actin-dependent drifts on filopodia and microvilli, diffusion and confinement. Virus transmission: drifts on actin-based extensions between infected and uninfected cells.	[2],[38]
Human immunodeficiency virus type 1 (HIV-1)	Retroviruses, lentivirus	CD4	CCR5, CXCR4 (chemokine receptors)	As reported for ALV	[2],[38]
Murine leukemia virus (MLV)	Retroviruses, ecotropic γ	mCAT1 (A-tropic: Pit-2, 10A-1: Pit-1, X-tropic and P-tropic: XPR)	?	As reported for ALV	[2],[38]
Human papillomavirus 16 (HPV16)	Papillomaviruses	Syndecan heparan sulfate proteoglycans, GPI-linked proteoglycans	?	Actin-dependent drifts on filopdodia, confinement	[72]
Murine poliomavirus-like particles (mPy-VLPs)	Polyomaviruses	Glycolipid gangliosides GD1a, GT1b	?	Diffusion, actin-dependent drifts, confinement	[23]
Simian virus 40 (monkey SV40)	Polyomaviruses	GM1 ganglioside	?	Diffusion, drifts, confinement, raft-dependent uptake	[26],[31],[111]
Adenovirus type 2 (Ad2)	Adenoviruses	CAR (coxsackievirus B adenovirus receptor)	αv β3/5 integrins	Diffusion, drifts, confinement	[11]
Vaccinia virus	Poxviruses	?	?	Virus entry: actin-dependent drifts on filopodia, confinement. Virus egress: propulsion by actin comet tails.	[55],[109]
Coxsackievirus B3	Picornaviruses	DAF (CD55, decay acceleration factor)	CAR (coxsackievirus B adenovirus receptor)	Apical targeting to tight junctions	[88]
Reovirus	Reoviruses	JAM-A (junction adhesion molecule)	β1 integrin	Confinement, waiting for clathrin-coated pits to appear	[95]
Influenza virus X31	Orthomyxoviruses	Sialic acid	?	Slow drifts, induction of clathrin-coated pits	[98]
Dengue virus	Flaviviruses	Mannose receptor	?	Slow diffusion	[93],[112]

?, unknown.

## Plasma Membrane Models Accounting for Heterogeneity

A large series of experimentations had shown earlier that the plasma membrane is not a homogeneous sheet of proteins and lipids (see e.g., [12],[13],[14]). In fact, membranes are organized into domains of ordered structures held together by cooperative molecular interactions between their constituents in a liquid environment [10]. For membrane domains of the size of viruses, that is, dozens to hundreds of nanometers in diameter, two nonexclusive models have been put forward, the fencing model and the “lipid raft” model. The fencing model suggests that membrane domains are bordered by the underlying cytoskeletal network, predominantly the cortical actin filaments (F-actin) [15]. This confines plasma membrane proteins and lipids to corrals where movement occurs more or less without restrictions [16],[17]. Switching of components between corrals occurs by hop diffusion.

The lipid raft model proposes that the movement of proteins in the lipid bilayer is constrained by the chemical composition of the membrane [18]. The primary components of biological membranes are glycolipids, cholesterol, and phospholipids, including glycerophospholipids and sphingomyelin [19]. Short unsaturated acyl chains increase membrane fluidity by weaker interactions between each other compared to sphingolipids. Unlike glycerophospholipids, the acyl chains of sphingolipids are typically saturated and longer, which increases their packing density in the bilayer. Cholesterol molecules further increase this ordered state, and give rise to so-called lipid rafts. Lipid rafts occur in the plasma membrane, endocytic membranes, and late secretory membranes, and incorporate certain proteins, such as glycosyl-phosphatidyl-inositol (GPI)-anchored proteins and double acylated tyrosine kinases, for example, of the Src family, or exclude others [18]. Although the precise size and composition of these rafts have been difficult to study [10], it is likely that the lipid microenvironment of the plasma membrane favours specific protein–protein interactions. For example, studies of the lipidome of human immune deficiency virus (HIV) and murine leukemia virus (MLV) recently showed that purified viruses contain an enriched set of unusual sphingolipids that are important for infection [20],[21]. This provides evidence that lipid domains do exist in cells, and actively participate in specific functions. Functional coordination of lipid domains with the underlying cortical actin network is likely to occur [22]. This would then give rise to spatial and temporal organization of lipid-tethered proteins as a result of the activity of the cortical actin network, and properties of the lipids.

## Surface Motions of Lipid-Attached Viruses

Studies of viruses attaching to lipid receptors provide strong evidence that lipid domains are involved in specific types of cell surface motions, as shown for example with murine polyomavirus (mPy)-like particles [23]. mPy is a small nonenveloped DNA tumor virus that uses glycolipid gangliosides GD1a and GT1b as receptors [24],[25],[26]. Unlike lipid raft domains, which are often immobile, such as caveolin-positive domains [27], mPy actively moves on cultured mouse fibroblasts in rapid random motion, in confined motions with constant drifts, and in confined motions within 30–60 nm zones [23]. Drifts and confined motions are actin-dependent, possibly mediated by cortical actin. The confinement of mPy on the surface lasts for minutes and delays endocytosis, implying that viral uptake requires the particle mobility. Particle mobility and infection crucially depend on the native membrane composition and fluidity, as concluded from depleting plasma membrane cholesterol, which completely immobilizes mPy and blocks infection.

Simian virus 40 (SV40) also uses glycolipids as a cell surface attachment site [26]. SV40 is a nonenveloped DNA virus of the papova (papilloma and polyoma) virus family. The particles are 45–50 nm in diameter and consist of viral proteins 1 to 4 (VP1–4). The major capsid protein VP1 is arranged as 72 pentamers in a T = 7D icosahedral lattice [28]. SV40 uses GM1 ganglioside as a receptor, and is taken up by caveolar and noncaveolar membrane domains [29]. Interestingly, the free ganglioside receptors are several orders of magnitude more mobile than the virus particles bound to the receptors in lipid bilayers [30]. This supports the notion that virions bind strongly and multivalently to cell surface glycolipids, in agreement with a recent crystal structure of VP1-GM1, which showed that each VP1 binds one GM1 [31]. Multiple receptor binding may impose constraints on both the viral capsid and the membrane, which can modulate the mobility of virus–receptor complexes and cell signalling [29],[32],[33],[34],[35],[36].

## Diffusion of Viruses on the Cell Surface

Diffusion of lipids and proteins in the plasma membrane is driven by thermal motion. Tracking of single fluorescent SV40 particles revealed that SV40 particles on the plasma membrane randomly diffuse shortly after warming and are then immobilized on caveolin-GFP patches [37]. This may suggest that virus diffusion occurs when low levels of receptor are bound to the particle, and is terminated when sufficiently high amounts of receptors are bound. Diffusion-based movements allow both lipid- and protein-bound viruses to scan several micrometers of cell surface within a few seconds, and thereby may search for sites that are competent for endocytosis or downstream signalling [11],[23],[38]. Although periods of diffusion are prevalent shortly after a virus has contacted the cell surface, they also occur with particles that have previously been engaged in other types of surface movements (Figure 1). This suggests that virus–receptor interactions on the surface are complex, and controlled by both intracellular and extracellular factors.

## Drifting Motions Occur by Coupling Plasma Membrane Receptors to Retrograde F-Actin Flow

**Figure 2:**
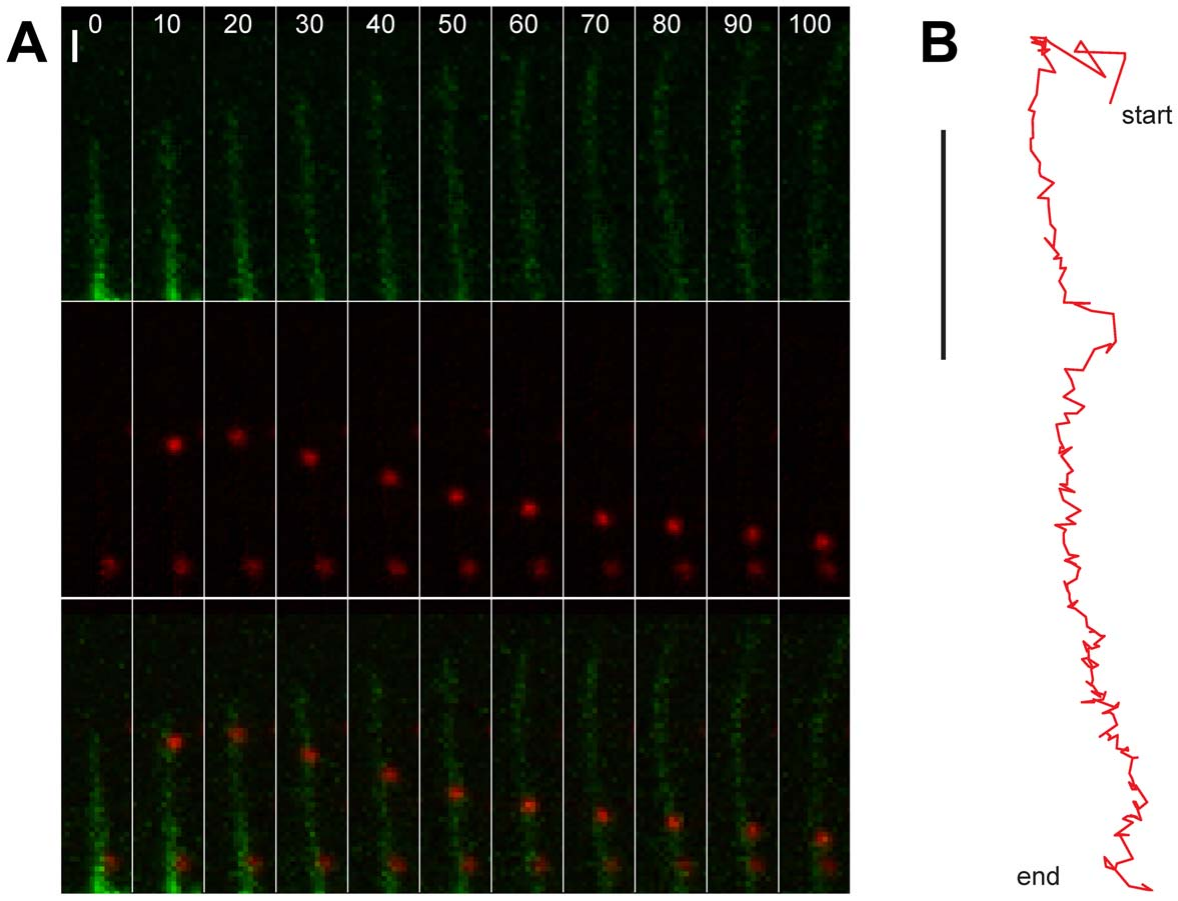
Viral surfing on a growing filopodium. (A) Red fluorescent human adenovirus type 2 particles (red puncta in middle and lower rows) and actin were imaged by spinning disc confocal microscopy [86] on human embryonic retinoblast 911 cells stably expressing GFP-actin (green structures in upper and lower rows). Note that the upper particle attached to a filopodium at time point 10 s, and engaged in a drifting motion towards the cell body (lower side of the images, not shown). During this movement, the filopodial actin structures expanded away from the cell body. A second virus particle bound to the same filopodium remained stationary up to 100 s, indicating that it was not coupled to the actin flow. Bar  = 2 µm. (B) Trajectory profile of the drifting particle from (A) acquired by automated tracking of 2 Hz images. Note that this particle covered approximately 7 µm in 90 s from the start point (10 s) to the end point (100 s) with an average speed of 0.08 µm/s Bar  = 2 µm.

Extracellular particles take advantage of directional movements inside cells by coupling to retrograde flow of F-actin (Figure 2). Early observations of dynamic processes in growth cones of neuronal cells had shown that F-actin can flow rearward in the form of ruffling waves [39], or as parallel bundles in filopodia [40], depending on actin treadmilling [41] and actin-based motors, such as myosin (Myo) II [42]. Membrane ruffles and filopodia are crucial for cell movements and formation of cell patterns [43],[44]. Ruffles are induced in response to extracellular stimuli by rapid actin polymerization. It requires the small GTPase Rac and downstream targets, such as WAVE proteins, which activate the actin nucleator Arp2/3. Filopodia are cell protrusions with terminal adhesion sites that allow migratory cells to explore extracellular space. Filopodia acquire their dynamics by treadmilling actin filaments, that is, actin monomers rapidely polymerize at the distal plus ends of actin filaments and depolymerize upon mechanical constraints, and by severing in the contraction zone of the cell body [45]. Experiments with antibody-coupled beads attached to the cell surface receptor apCAM on growth cones of *Aplysia* neurons demonstrated that retrograde flow required clustering of the receptors and signal transduction [46]. Using fluorescence speckle microscopy in combination with chemical inhibitors of Myo II and actin polymerization, it was shown that retrograde flow is a steady state that almost entirely depends on both Myo II contractility and actin-network treadmilling, thereby supporting cell migration [45].

**Figure 3:**
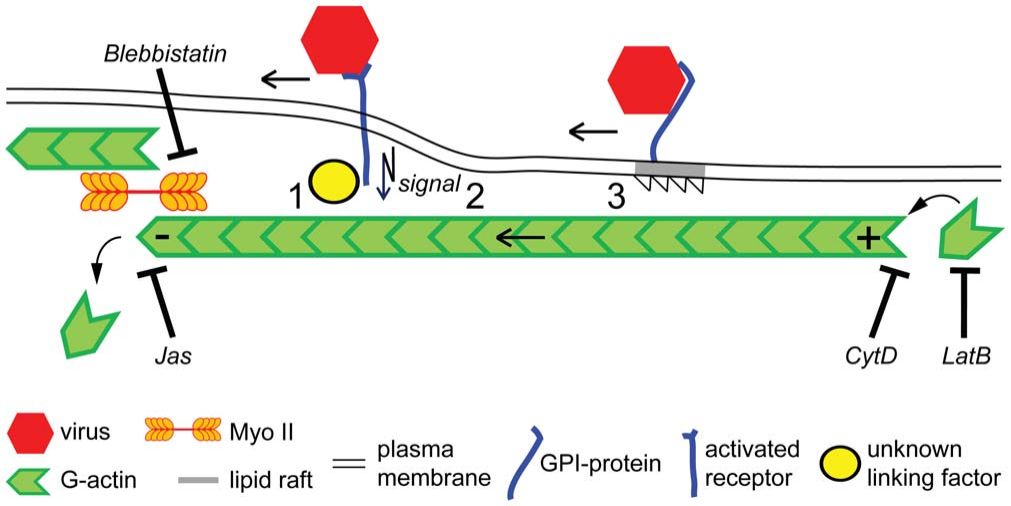
Principles of virus coupling to retrograde actin flow. Retrograde flow of filamentous actin (F-actin) is maintained by two machineries. One is actin filament polymerization at the plus end of the filament, for example, near the tip of a filopodium, and depolymerization at the opposite minus end. Depolymerization of F-actin by cytochalasin D (CytD), inhibition of actin polymerization by latrunculin B (LatB), or stabilization by jasplakinolide (Jas) inhibit retrograde flow of F-actin, virus drifts on filopodia, and also infection. The second machinery is based on the myosin II (Myo II) motor, which pulls actin filaments to the cell body. Myo II is anchored in the actin mesh at the cell body and cortex. Inhibition of Myo II by blebbistatin inhibits actin retrograde flow, virus drifts, and infection. The linkage of viruses to retrograde flow can occur through viral transmembrane receptors directly or indirect to F-actin (1), or require signalling downstream of virus binding and receptor clustering (2). Another mechanism is by the partitioning of receptors into specialized membrane domains, such as lipid rafts that transiently link to actin retrograde flow (3).

A direct mechanical link between transmembrane receptors and actin filaments was suggested for retrograde flow of the cell adhesion molecule L1-CAM (for illustration, see Figure 3). L1-CAM engages with retrograde F-actin flow, but is also diffusive or stationary, consistent with interactions between the receptors and different cytoskeletal proteins. For example, L1-CAM interacts with the actin adaptor proteins ankyrin and ezrin [47], which leads to stationary behaviour of the receptor, and restricts L1-CAM-mediated axon growth [48]. These constraints generate a traction force that was found to be regulated by phosphorylation of L1-CAM [49].

Another mechanism to couple receptors to the F-actin flow is through the tensile forces resisting the drag forces in the hydrodynamic flow at the cell surface [50],[51]. Interestingly, the strength of receptor coupling to actin was found to depend on the extent of extracellular force [46],[52]. Although the force sensors are unknown, a mechanical coupling mechanism could be widespread [53], and may be used by viruses.

## Drifting Motions Mediate Viral Transmission between Cells

**Figure 4:**
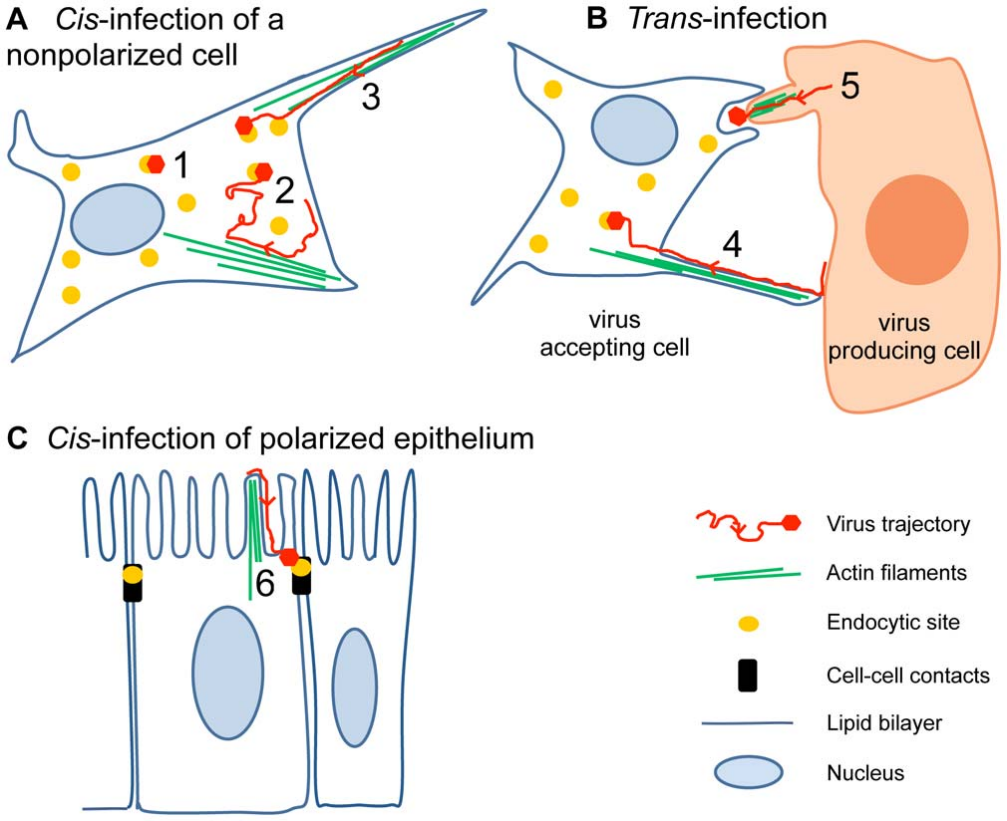
Infectious lateral mobility of viruses on the cell surface. (A) *Cis*-infection by virus targeting to endocytic hot spots. Reovirus, for example, depends on clathrin-coated pits that form near the virus [95] (yellow dots, scenario 1). Other viruses, such as influenza virus, induce their own clathrin-coated pits [98]. Polyomaviruses [37], papillomaviruses [72], or dengue virus [93] may use various types of motions to scan the surface for preexisting coated pits or caveolae (2). Retroviruses [2],[38], papilomavirus [72], vaccinia virus [55],[109], adenovirus [11], and polyomaviruses [23] use directional drifts from the distal tips of filopodia to the cell body (3). (B) *Trans*-infection by cell surface movements. Cell-to-cell transmission of extracellular retroviruses or herpesviruses can occur in virological synapses and cytonemes from the surface of a donor cell to an acceptor cell [62],[110] (4). Vaccinia virus egress is driven by actin comet tails that form underneath an extracellular virus, and thereby propel the virus towards an acceptor cell [109] (5). (C) Virus infection of epithelia. Coxsackievirus B, an enterovirus of the picornavirus family, is targeted to cell–cell contacts (black bars), where it interacts with the endocytic machinery (yellow dot) [88] (6). Retroviruses move along microvilli to reach the cell body, where they may be endocytozed or fuse with the plasma membrane [60].

F-actin-dependent motion of viruses on the cell surface was initially observed with the retroviruses MLV and avian leukosis virus (ALV) on actin-rich microvilli and filopodia [38]. Filopodia are prominent in cortical neurons, and antigen-presenting cells, such as dendritic cells or macrophages, and are involved in viral infections and cell–cell transmission of viral particles (Figure 4). The envelope (env) protein of MLV binds to the mouse cationic amino acid transporter-1 (mCAT-1), which leads to receptor clustering [38]. Viruses are pulled towards the cell body by actin polymerization and Myo II. It was suggested that this supports infection by moving virus particles retrogradely to sites on the cell body that are particularly competent for endocytic uptake (Figure 4A and 4B).

F-actin flow, unlike diffusion, may also allow viruses to break free from nonproductive confinements and may couple actin dynamics to signalling and endocytosis. This is supported by the observation that certain forms of vaccinia virus from the poxvirus family, the so-called MVs (intracellular mature viruses), are transported retrogradely on filopodia to the cell body where they induce actin turnover and membrane blebbing [54],[55]. MVs are assembled in the cytoplasm by wrapping the DNA-containing capsid with a single membrane that is probably derived from the endoplasmic reticulum (ER) [56]. Signalling by extracellular MVs activates p21-activated kinase 1 and leads to the formation of macropinosomes, similar to macropinosome induction by human adenovirus [57], echovirus 1, or epidermal growth factor (EGF) [58]. It is possible that retrograde motion of vaccinia virus involves a signaling receptor, since many poxviruses express EGF-like growth factors that target ErbB-1, and infection of animals can be blocked with ErbB-1 inhibitors [59].

Retrograde F-actin flow also increases the dispersion of viral particles from infected cells to noninfected cells and thereby enhances infection. This has been demonstrated with MLV in cell culture experiments where env-expressing infected cells attach to mCAT-1 of filopodia from neighboring noninfected cells [2]. Filopodial bridges, also called cytonemes, are similarly used by HIV or herpesviruses as highways to access noninfected cells [60],[61].

Retrograde flow on filopodia is particularly important for viral transmission between polarized cells of respiratory or intestinal epithelia and immune cells, and depends on receptor clustering induced by the multivalent pathogen [62]. This may complement transmission events in the “virological synapses” (Figure 4B). Virological synapses are sites of cell–cell contacts where viruses, such as HIV, human T cell leukemia virus, or herpesviruses are endocytozed and regurgitated, or directly transmitted to noninfected cells (for recent discussions of the two models, see [3],[63]). In the case of HIV, the env glycoprotein gp120 binds and clusters the CD4 receptors and the CCR5 or CXCR4 coreceptors [64]. Subsequent movement of virus particles requires the actin crosslinking protein filamin-A, which tethers CD4 to F-actin, and cofilin activation via the RhoA GTPase and the Rho kinase ROCK to induce actin dynamics and treadmilling [65]. Interestingly, this cascade leads to activation of the viral envelope protein gp41, which mediates fusion between the viral and the cellular membranes. In addition, interaction between gp120 and the co-receptor CXCR4 triggers cell signaling and activates cofilin, a major regulator of actin dynamics [66]. Activated cofilin enhances F-actin depolymerization, relieves the cortical actin barrier, and enhances entry of the viral capsid into the cell. An interesting challenge now is to determine to what extent the motions that have been recorded in cultured cells contribute to infections of organisms. For example, HIV inoculation into a human cervicovaginal organ culture system has already shown that infectious viruses attach to mucus-free regions of the cervical epithelium [67].

## Cell–Cell Transmission of Viruses by Actin Polymerization

Poxviruses are large enveloped DNA viruses and are pathogenic to humans and animals. The best studied prototypic strain is vaccinia virus, which infects a large variety of cell types from many different organisms. Two predominant forms of poxviruses are found in the cytosol, MVs and intracellular enveloped viruses (IEVs). Unlike MVs, IEVs contain two membranes that are derived from Golgi or endosomal membranes [68]. IEVs are transported by kinesin motors on microtubules to the cell periphery where they fuse their outer membrane with the plasma membrane [4]. The viruses that remain attached to the cell, the cell-associated enveloped viruses (CEVs), signal back to the cell by engaging the envelope protein B5R to an unknown receptor. This activates the tyrosine kinase Src, which is required to phosphorylate the cytoplasmic tail of the viral transmembrane protein A36R [69]. Tyrosine kinase activation leads to the formation of actin tails that propel the virions away from the cell body towards neighboring cells (Figure 4B). Actin tail formation and CEV motility and detachment require different tyrosine kinases, and this gives rise to extracellular enveloped viruses (EEVs) [70]. Blocking tail formation strongly reduces the spreading of infection. EEVs infect neighboring cells in the absence of signalling [54], which may in part explain why vaccinia virus induces relatively little inflammation in the respiratory tracts [71].

## Drifting Motions of Nonenveloped Viruses

The first nonenveloped virus shown to use retrograde F-actin flow for infection was human papilloma virus type 16 (HPV16) [72]. Among the high-risk papilloma viruses, HPV16 is a major cause of cervical cancer [73]. This virus infects basal differentiating keratinocytes of mucosal tissue, preferably in a wounded epithelium [74]. It binds to heparan sulfate proteoglycans, such as transmembrane syndecans, or GPI-linked proteoglycans, and additional receptors [75],[76],[77]. Processive movements of HPV16 virus-like particles (VLPs) were observed on filopodia at rates similar to a retrograde F-actin flow of 1–5 µm/min [72]. These movements supported infection and were inhibited by actin depolymerizing or stabilizing agents, or inhibitors of Myo II, myosin light chain kinase, or ATP synthesis, suggesting that active processes control actin filament turnover and retrograde movements.

Although it is not known how HPV16 couples to F-actin flow, the coupling mechanism may depend on the flow strength since particles alternated between drifting and confinement. This could, for example, involve a hierarchical slippage clutch that would mediate “frictional coupling” and differential transmission of F-actin–based forces through a network of transient protein–lipid or protein–protein interactions. Such mechanisms could be similar to F-actin flow in focal adhesions [51]. Interestingly, both moving and stationary HPV16 VLPs can be observed next to each other on single protrusions [72]. This suggests that the stationary viruses are trapped by retention and resist membrane flow, or that they are not coupled to F-actin flow as suggested for L1-CAM [48]. Yet, other virus particles were found in random motions hours after inoculation. It is unknown at present whether HPV16 uses the syndecan receptors for random movements, or cell adhesion receptors for coupling to the F-actin flow.

Compared to polyomaviruses, adenoviruses are 2-fold larger and more amenable for single particle tracking. They can be labelled with hundreds of fluorophore molecules, which makes them extremely bright point sources of light, ideal for tracking at high spatial and temporal resolutions [11],[78]. Adenoviruses infect the upper and lower respiratory tracts, the urinary and digestive tracts, lymphoid systems, and heart, and give rise to epidemic conjunctivitis [79],[80],[81]. They account for approximately 7% of respiratory virus infections in humans [82],[83]. Adenovirus type 2 (Ad2) binds to the immunoglobulin superfamily protein coxsackie and adenovirus receptor (CAR), and interacts with alpha v integrins before clathrin and dynamin-mediated endocytosis [84],[85]. Single particle tracking suggests that Ad2 movements on the cell surface lead the virus particles to plasma membrane domains proficient for endocytosis, or recruit endocytic effector proteins while they are in a particular motion mode [11],[86].

## Coupling Surface Motions to Polarized Virus Entry

How viruses enter into polarized cells is a question of major importance, and has been addressed with several cell culture models [87]. An interesting connection between actin dynamics and polarized entry was found for coxsackie virus B3 (CVB) in differentiated human intestinal CaCo2 cells [88]. CVB3 is a nonenveloped RNA enterovirus that binds to the GPI-anchored decay accelerating factor (DAF, CD55) at the apical plasma membrane. DAF is an important inhibitor of the complement cascade, and blocks the C3 convertase on the apical membrane. Upon attachment of CVB3 to DAF, DAF is cross-linked and membrane domains are sequestered into lipid rafts. Coincidentally, the tyrosine kinase c-Abl is activated, and the actin cytoskeleton reorganized, leading to CVB3 targeting to tight junctions between polarized cells. The Abl inhibitor Gleevec blocked CVB3 targeting to the junctions and reduced infection. Whether CVB3 uses active actin-dependent transport of DAF on microvilli, or diffusion in the membrane, is not known, however. Tight junction targeting of CVB3 is, however, crucial for the virus to access the secondary receptor CAR (Figure 4C). CAR is an entry and uncoating receptor for CVB, upstream of viral endocytosis [84],[89]. Virus engagement with CAR destabilizes the capsid and exposes VP4, which is involved in pore formation in the limiting endosomal membrane and faciliates RNA release to the cytosol [90]. Blocking CVB from reaching the tight junctions provides a mechanism for interference with the host to inhibit infection.

## Confinement of Viruses to Endocytic Spots

Clathrin-mediated endocytosis is a deeply characterized endocytic pathway, and has been linked with particular motions of viral particles on the cell surface. It delivers ligand-receptor complexes to early endosomes and other vesicular compartments, including late endosomes, recycling endosomes, and Golgi membranes [91],[92]. Cell biological experimentation indicated that there are two populations of clathrin-coated pits, static and dynamic pits, which seem to have distinct functions for viral infections. For example, dengue virus appears to visit preexisting static clathrin-coated pits, and is delivered to late endosomes where it fuses with the limiting membrane [93]. Clathrin-coated pits have a distinct but limited actin-dependent mobility in the plasma membrane [94], which implies that they can assemble and disassemble at variable sites on the plasma membrane [95]. Mobile clathrin-coated pits seem to be involved in Semliki Forest virus infection and deliver viruses to early endosomes [96]. It has been suggested that certain viruses, such as reovirus, randomly engage with clathrin-coated pits and stabilize the pits, which leads to confinement of the pit and the virus [95]. The confined virus then traffics to a cathepsin-positive compartment, presumably late endosomes and lysosomes [97]. Influenza viruses and Semliki Forest virus may use both preexisting and newly assembled clathrin-coated pits for entry and transport to early and late endosomes [96],[98].

In contrast to large viral cargo, transferrin, an iron carrier protein of about 5 nm, is constitutively internalized by short lived clathrin-coated pits within less than one minute, implying that the rate of clathrin-coated pit formation either depends on the size of the ligand, or functional differences in the clustering mechanisms. The latter could be due to signalling from clustered viral receptors, in analogy to the G protein-coupled receptor (GPCR) delta-opioid. This receptor delays its agonist-stimulated uptake from clathrin-coated pits by PDZ-dependent linkage to the actin cytoskeleton [99]. Interestingly, the recruitment of the large GTPase dynamin, which controls clathrin-mediated endocytosis, is delayed in this case. The formation of the receptor-β-arrestin complex and association with preexisting clathrin-coated pits are not affected, suggesting that receptor uptake is inhibited at a late stage of clathrin-coated pit formation. An increased surface residence time of GPCR-β-arrestin complexes is thought to enhance mitogenic signalling, unlike the β-arrestin-free GPCR.

Whether functional specialization of clathrin-mediated endocytosis leads to the generation of distinct endosomes or endosomal domains is an open question [100]. It has been suggested that distinct cargoes of the clathrin pathway are differentially sorted into different types of early endosomes, and that such events are initiated at the plasma membrane [101]. For example, human influenza A viruses bind to terminal sialic acid moieties of glycoproteins and glycolipids on nonciliated cells of the upper airways [102]. In particular, the influenza A strain X-31 (H3N2) engages in slow actin-dependent motions on BS-C-1 monkey kidney cells, and induces its own clathrin-coated pits before internalization into the fast maturing endosomal pathway [98]. Collectively, these data suggest that different virus–receptor pairs engage in different ways with clathrin-coated pits, and can be targeted to distinct intracellular sites.

## Conclusions

Evidence from single particle tracking experiments has demonstrated that particular motion types on the cell surface support infection. These motions include diffusion, drifting motions, and confinement of virus–receptor complexes. Recent data from a variety of unrelated viruses indicate that directional F-actin flow is a powerful gate into cells. It also facilitates transmission of infectious virus particles between infected and noninfected cells, for example by supporting *trans*-infection between immune cells and T cells. We expect that most viruses will be found to use retrograde F-actin flow, if they bind to specific cell surface receptors. In instances of nonspecific attachment, for example, viruses binding to heparan sulfate proteoglycans, which are interlinked with plasma membrane proteins or the extracellular matrix, we expect the virus particles not to drift, until they attach to a receptor connected to the plasma membrane. Evidence for this has come from single virus tracking experiments of HIV VLPs in cultured cells [103].

It will be of great importance to define the specific virus–receptor complexes and the membrane domains that support viral surface movements. We already know, for example, that different receptors have different attachment mechanisms to cortical F-actin flow. Specifically, receptor linkages to F-actin can be regulated or stochastically determined, and they can be direct, through adaptor proteins, or indirect through a series of low affinity interactions of clustered protein or lipid receptors. The mechanisms of how particular virus–receptor complexes link to F-actin flow have implications on how long the viruses stay in the drifting mode, and how they recruit effector proteins for downstream events. This is important for viral interactions with immune cells and polarized epithelial or neuronal cells. Future analyses will identify how distinct motion types connect to the infectious endocytic uptake processes, or the noninfectious processes leading to virus destruction or immune presentation.

## Perspectives for Anti-Viral Strategies

The concept of targeting the host for anti-viral therapy was introduced in the 1990s by giving hepatitis C virus–infected individuals interferon-alpha in combination with ribavirin [104],[105]. This paradigm is currently extended in the infectious disease community by systematic profiling of host genes using transcriptome and RNA interference, in combination with cell biological, single virus particle tracking, and bioinformatics studies. Several interesting observations have come from such studies. For example, nonreceptor tyrosine kinases of the src family control the dynamics of actin during egress of vaccinia virus from or entry of coxsackie virus or enteropathogenic bacteria into the cell (for a review, see [69]). This is an important advance towards applying small chemicals against the host to inhibit infection. The small compound Gleevec (Imatinib mesylate, STI571), which inhibits the tyrosine kinases c-Abl and c-Kit, is licensed for the treatment of chronic myelogenous leukemia, and blocks infections of cultured cells and mice with poxviruses, coxsackie viruses, or enteropathogenic bacteria [88],[106]. Gleevec also interferes with Kaposi's sarcoma herpesvirus infections of cultured cells [107], and reduces the tumor mass of Kaposi's sarcoma patients [108]. The development of new classes of agents blocking virus motions on the cell surface could extend the concept of host interference against infection, distinct from receptor targeting strategies, which are prone to rapid emergence of viral resistance.
